# Benchmarking of survival outcomes following Haematopoietic Stem Cell Transplantation (HSCT): an update of the ongoing project of the European Society for Blood and Marrow Transplantation (EBMT) and Joint Accreditation Committee of ISCT and EBMT (JACIE)

**DOI:** 10.1038/s41409-023-01924-6

**Published:** 2023-03-09

**Authors:** Riccardo Saccardi, Hein Putter, Dirk-Jan Eikema, María Paula Busto, Eoin McGrath, Bas Middelkoop, Gillian Adams, Marina Atlija, Francis Ayuketang Ayuk, Helen Baldomero, Yves Beguin, Rafael de la Cámara, Ángel Cedillo, Anna María Sureda Balari, Christian Chabannon, Selim Corbacioglu, Harry Dolstra, Rafael F. Duarte, Rémy Dulery, Raffaella Greco, Andreu Gusi, Nada Hamad, Michelle Kenyon, Nicolaus Kröger, Myriam Labopin, Julia Lee, Per Ljungman, Lynn Manson, Florence Mensil, Noel Milpied, Mohamad Mohty, Elena Oldani, Kim Orchard, Jakob Passweg, Rachel Pearce, Régis Peffault de Latour, Hélène A. Poirel, Tuula Rintala, J. Douglas Rizzo, Annalisa Ruggeri, Carla Sanchez-Martinez, Fermin Sanchez-Guijo, Isabel Sánchez-Ortega, Marie Trnková, David Valcárcel Ferreiras, Leonie Wilcox, Liesbeth C. de Wreede, John A. Snowden

**Affiliations:** 1 Cellular Therapy and Transfusion Medicine Unit, Careggi University Hospital, Florence, Italy; 2 Department of Biomedical Data Sciences, Leiden University Medical Center, Leiden, the Netherlands; 3 EBMT Statistical Unit Leiden, Leiden, the Netherlands; 4 EBMT Registry, Leiden, the Netherlands; 5 ICCBBA, Barcelona, Spain; 6 EBMT Executive Office, Barcelona, Spain; 7 Department of Stem Cell Transplantation, University Medical Center Hamburg, Hamburg, Germany; 8 Centre for Hematology and Oncology, University Hospital Basel, Basel, Switzerland; 9 Department of Hematology, CHU of Liège and University of Liège, Liège, Belgium; 10 Infectious Diseases Working Party (IDWP), Barcelona, Spain; 11 Grupo Español de Trasplantes Hematopoyéticos y Terapia Celular (GETH-TC), Madrid, Spain; 12 Hematology Department, Institut Català d’Oncologia-Hospitalet, Barcelona, IDIBELL, Universitat de Barcelona, Barcelona, Spain; 13 Centre de Thérapie Cellulaire, Institut Paoli-Calmettes Comprehensive Cancer Centre, Aix-Marseille Université School of Medicine & Inserm CBT-1409, Centre d’Investigations Cliniques en Biothérapies, Marseille, France; 14 Department of Paediatric Oncology, Haematology and Stem Cell Transplantation, University Children’s Hospital Regensburg, Regensburg, Germany; 15 Laboratory of Hematology, Department of Laboratory Medicine, Radboud University Medical Center, Nijmegen, the Netherlands; 16 Hospital Universitario Puerta de Hierro Majadahonda, Universidad Autónoma de Madrid, Madrid, Spain; 17 Sorbonne Université, INSERM UMRs938, Service d’Hématologie Clinique et de Thérapie Cellulaire, Hôpital Saint Antoine, AP-HP, Paris, France; 18 Blood and Marrow Transplant (BMT) Unit, IRCCS San Raffaele Hospital, Milan, Italy; 19 Autoimmune Diseases Working Party (ADWP), EBMT, Barcelona, Spain; 20 Department of Haematology, St Vincent’s Hospital Sydney, Sydney, NSW Australia; 21 School of Clinical Medicine, UNSW Medicine & Health, Kensington, NSW Australia; 22 School of Medicine, Sydney, University of Notre Dame Australia, Sydney, WA Australia; 23 Kenyon, M, Department of Haematology, King’s College Hospital NHS Foundation Trust, London, United Kingdom; 24 Sorbonne University, Department of Hematology, Hôpital Saint Antoine, and INSERM UMRs 938, Paris, France; 25 EBMT Paris Study Office, Paris, France; 26 British Society of Blood and Marrow Transplantation and Cellular Therapy (BSBMTCT) Data Registry, London, United Kingdom; 27 Department of Cellular Therapy and Allogeneic Stem Cell Transplantation, Karolinska University Hospital, Division of Hematology, Department of Medicine Huddinge, Karolinska Institutet, Stockholm, Sweden; 28 Edinburgh & SE Scotland Clinical Transfusion Centre Patient Services and the Edinburgh & SE Scotland Clinical Apheresis Unit, Edinburgh, United Kingdom; 29 Agence de la Biomédecine, Saint-Denis, France; 30 Centre Hospitalier Universitaire (CHU) Bordeaux, Service d’Hematologie et Therapie Cellulaire, Bordeaux, France; 31 Sorbonne University, Hôpital Saint Antoine (AP-HP), and INSERM UMRs 938, Paris, France; 32 Italian National BMT Registry (GITMO), Bergamo, Italy; 33 University Hospital Southampton NHS, London, United Kingdom; 34 Hematology division, Basel University Hospital, Basel, Switzerland; 35 BMT unit Paris Cité University, French reference center for aplastic anaemia and PNH, French rare network for rare immunological and haematological disorders (MaRIH), Severe aplastic anaemia working party of the European blood and marrow transplantation group (SAAWP EBMT) Saint-Louis Hospital, Paris, France; 36 Belgian Transplant Registry, Belgian Cancer Registry, Brussels, Belgium; 37 King’s College Hospital NHS Foundation Trust, London, United Kingdom; 38 CIBMTR, Medical College of Wisconsin, Milwaukee, WI USA; 39 Hematology and Bone Marrow Transplantation Unit, IRCCS San Raffaele Scientific Institute, Milano, Italy; 40 JACIE, EBMT Executive Office, Barcelona, Spain; 41 CIC-University of Salamanca, IBSAL-University Hospital of Salamanca, Salamanca, Spain; 42 Czech registry, Prague, Czech Republic; 43 Hematology Department, Vall d’hebron Institut Oncologic (VHIO), Hospital Vall d’hebron, Universitat Autònoma de Barcelona, Barcelona, Spain; 44 Australasian Bone Marrow Transplant Recipient Registry (ABMTRR), Darlinghurst, NSW Australia; 45 Department of Haematology, Sheffield Teaching Hospitals NHS Foundation Trust, Sheffield, United Kingdom

**Keywords:** Haematological cancer, Therapeutics

## Abstract

From 2016 EBMT and JACIE developed an international risk-adapted benchmarking program of haematopoietic stem cell transplant (HSCT) outcome to provide individual EBMT Centers with a means of quality-assuring the HSCT process and meeting FACT-JACIE accreditation requirements relating to 1-year survival outcomes. Informed by previous experience from Europe, North America and Australasia, the Clinical Outcomes Group (COG) established criteria for patient and Center selection, and a set of key clinical variables within a dedicated statistical model adapted to the capabilities of the EBMT Registry. The first phase of the project was launched in 2019 to test the acceptability of the benchmarking model through assessment of Centers’ performance for 1-year data completeness and survival outcomes of autologous and allogeneic HSCT covering 2013–2016. A second phase was delivered in July 2021 covering 2015–2019 and including survival outcomes. Reports of individual Center performance were shared directly with local principal investigators and their responses were assimilated. The experience thus far has supported the feasibility, acceptability and reliability of the system as well as identifying its limitations. We provide a summary of experience and learning so far in this ‘work in progress’, as well as highlighting future challenges of delivering a modern, robust, data-complete, risk-adapted benchmarking program across new EBMT Registry systems.

## Introduction

HSCT is a complex medical procedure involving consideration of numerous biological and clinical components and outcomes are impacted by many factors, including patients, donors, disease and transplant characteristics. The field is constantly developing, for example with the introduction of innovative molecules for controlling and/or preventing relapse after the transplant [1–4], novel cellular therapies [5] and the growing use of alternative mismatched donors [6–8], which continually increase the complexity of clinical management and laboratory processes required for high-quality patient and donor care. Procedures also come with an economic cost in terms of healthcare resources, and there is a broader responsibility for transparency called for by different organizations, including patients’ associations, private insurance companies, accreditation bodies and health authorities [9, 10]. Benchmarking, as well as best practice management guidelines, is considered a reliable tool to improve the quality of clinical output and the proficiency in care delivery, while contributing to the financial sustainability of health care systems [11]. An international benchmarking project can provide new insights into different health systems and organizational models in HSCT practice across Europe and further afield and demonstrate the need to improve and harmonize in line with an agreed standard.

In 2016, the EBMT Board and the JACIE Committee launched a benchmarking project directed at EBMT transplant Centers. According to the FACT-JACIE standards the accreditation covers the entire transplantation process, from the selection of the donor/ patient to the follow-up, including collection, characterization, processing and storage of the graft. A Clinical Outcomes Group (COG) was established to review the existing national and international experience and to define the fundamentals of an international system for benchmarking of survival outcomes following HSCT. A statistical model was developed by the Department of Biomedical Data Sciences of the Leiden University Medical Center (LUMC) in collaboration with the EBMT Statistical Unit, taking into account case-mix correction including factors associated with disease risk, transplant technology and clinical characteristics of patients. The initial steps of the project were published in 2019 [12] and a ‘first phase’ was launched using the data extracted from the EBMT ProMISe database over a 4-year observation time period. The objectives were to test the statistical model, to analyse the data completeness in the Registry and to develop a reporting methodology for the EBMT programs selected for the project. National scientific societies and registries were also involved to facilitate project dissemination. In 2021 a ‘second phase’ was started, targeted at a 5-year observation period: a refinement of the case-mix factors and the data collection forms is currently ongoing. We report here an update on the project: in particular, the trend to join the project by EBMT centers and a descriptive comparison of data completeness in the two analyzed periods is reported.

## Methods

### Observation periods

During the first phase, the 2013–2016 timeframe was selected. The second phase observation period was a 5-year interval, from January 1st 2015 to December 31st 2019; the 1-year follow-up of patients transplanted by the end of 2019 was collected. Future benchmarking activities will follow the same schedule, that is: 5-year observation interval, minimum 1-year follow-up and data extraction in May of the following year. The latter allows Centers 4 months to update the follow-up of patients transplanted by the end of the observation period.

### Center selection

Transplant Centers were identified through their Center Identification Code (CIC), as extracted from ProMISe database. The criteria for patient and Center selection were agreed by the COG in the initial phase of the project and were maintained throughout [12]. In particular, the minimum number of transplants per year performed in the observed period was consistent with the JACIE accreditation requirements and adequate data reporting to the EBMT Registry was also required. The Center selection criteria are reported below:

- Full EBMT membership
- Transplants reported to the EBMT Registry >80% of the activity reported in the Activity Survey
- Allogeneic transplants: a minimum of 10 allografts/year on average in the observation period
- Autologous transplants: a minimum of 5 autografts/year on average in the observation period

Satisfactory clinical follow-up reporting is essential for fair and accurate benchmarking. Therefore, 1-year mortality was assessed only in Centers with a ratio of total observed to total potential follow-up higher than 80%. Centers that met the threshold for 1-year follow-up received their Clinical Outcomes assessment in the same report.

### Selection of cases

Only first allogeneic and autologous transplants were included in both phases, excluding solid tumors in allogeneic transplants and non-haematological diagnoses and paediatric patients in autologous procedures.

### Data extraction

The second analysis was carried out from data extracted from ProMISe on the 14th of May 2021. From this dataset, all first allogeneic and first autologous transplants performed in the period January 1st 2015 up to December 31st 2019 were analyzed. Patients who received a second transplant before 1 year were not censored for survival at time of the second transplant.

### Case-mix

The variables selected as covariates for case-mix adjustment were previously reported [12]. All data were included in the EBMT MED-A form, either as single values or composite scores to be calculated from the raw data. Two composite variables were included in the case-mix.

1. The Disease Risk Index (DRI) [13] is used as an independent disease risk classification system and is validated for allogeneic HSCT. Adjustments have been made based on the available data. In the case of Myelodysplastic Syndromes (MDS) stage, all except RAEB-1 and RAEB-2 and patients transformed to Acute Myeloid Leukaemia (AML) are classified as low risk MDS.
  The DRI requires information on cytogenetic abnormalities to risk-stratify MDS and AML. For MDS, the categorization follows Armand’s original categorization scheme [13]. For AML, cytogenetic classification is done according to the UK Medical Research Council (MRC) cytogenetic classification [14]. For either MDS or AML, cytogenetics was considered missing if no chromosome analysis (by any method) results were registered, or if chromosome analysis results were abnormal, with no further details registered.
  The original definition of the DRI did not account for the following diagnostic groups; bone marrow failure, inherited disorders, auto-immune diseases, histiocytic disorders and hemoglobinopathies. In the adjusted DRI used in benchmarking, these diagnoses were classified as ‘low risk’.
2. The Hematopoietic Cell Transplantation-specific Comorbidity Index (HCT-CI) is an independent patient-associated risk indicator, initially developed in the allogeneic setting [15] and then also reported in autologous HSCT [16, 17]. HCT-CI was calculated based on the individual comorbidities as reported in ProMISe: when a Center indicated that any comorbidity was present, at least one comorbidity needed to be entered. When some comorbidities were entered, the remaining comorbidities were assumed to be absent. Age-adjusted HCT-CI [18] was not used as adjustment for age is accommodated in the benchmarking statistical model.

DRI and HCT-CI were analysed through the same methodology in both the phases.

The complete list of covariates used in the prediction model for the allo- and auto benchmarks is reported in the Supplementary materials #2.

### Statistical methods and data completeness

A description of statistical methods was previously reported [12]; funnel plot model in benchmarking analysis was also detailed elsewhere [19]. Missing values in case-mix and other baseline patient characteristics were imputed, prior to the estimation of the follow-up and clinical outcomes benchmarks. Multiple Imputation by Chained Equations (MICE) [20, 21] was applied in case of missing case mix variable values; 5 imputation datasets were generated, each by 10 iterations. Outcome information was included as the Nelson-Aalen estimator and the event indicator (dead/lost to follow-up). This method provides unbiased estimation of the hazard ratios of the case-mix variables, which are then used to calculate the expected number of censored patients for FU or the number of dead patients for clinical outcomes.

After fitting the case-mix model for the actual benchmarking, a method of single value imputation is applied. Continuous variables are imputed by the median value and categorical values are imputed by mode, each among 12 month survivors only. Therefore, missing values are replaced by drawing from a relatively healthy sub-population, resulting in a lower value of the predicted probability of death, and subsequently a lower number of expected events in a Center increasing the ratio of observed and expected events upward. Generally, the higher the O/E ratio (observed over expected), the worse the Center’s performance within the benchmarking exercise. It is anticipated that this method of imputation should incentivise Centers to maximise their data completeness.

### Center reports

The report of each Center performance was made available to the local Principal Investigators (PI) in both.pdf and html format and the data concerning autologous and allogeneic transplants were kept separate. The report included an overview of the project and the methodology and an extended list of tables and plots describing both data completeness and Center performance.

### Report distribution

The Center PI was notified by e-mail that their report was available providing them with the instructions to download the individual report through a secure web-based drive (Google). Centers that were excluded were also notified with the reason for their exclusion. In the second phase, the Center’s data manager also received a separate notification that the report was available to increase awareness within the Center that the second exercise was underway and that their Center was included. When expressly authorized by the PI, the corresponding National Registry was also allowed access to the report in order to take advantage of their proximity to the Centers in their country.

### Center feedback

In both project phases, a questionnaire was circulated to the PI who had downloaded their report, to assess the interest in the project and to evaluate the utility and fairness of the reports, their accessibility and clarity.

## Results

### Patient cohorts

**Fig. 1:**
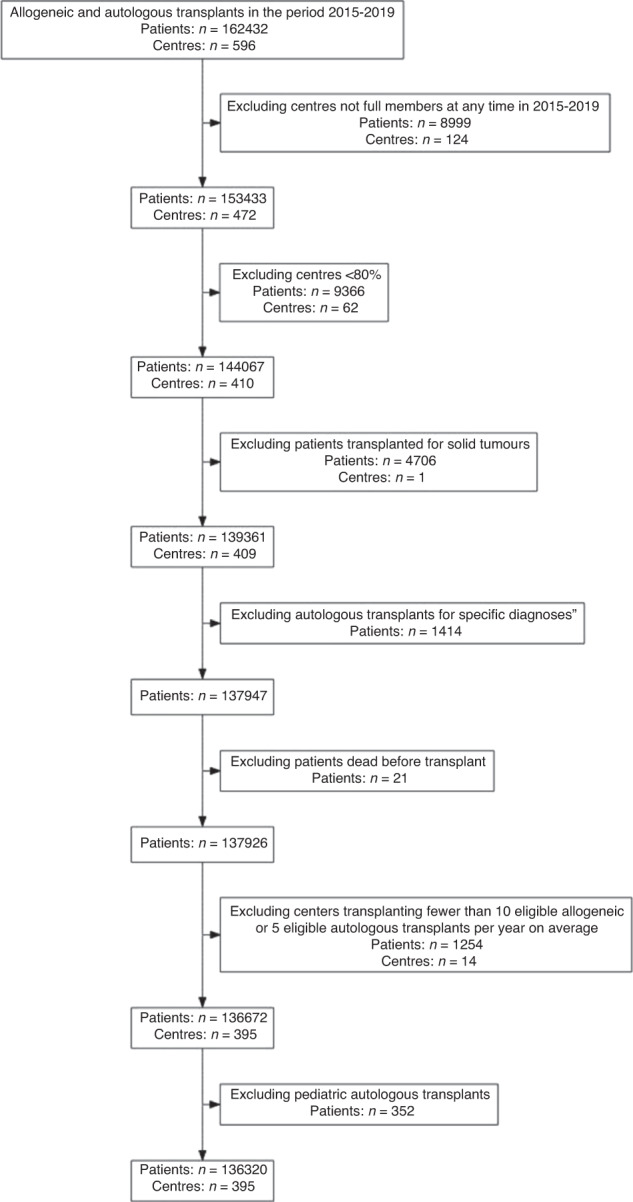
Patients selection. Selection process of patients to be analysed in the second phase of the Benchmarking Project.

**Fig. 2:**
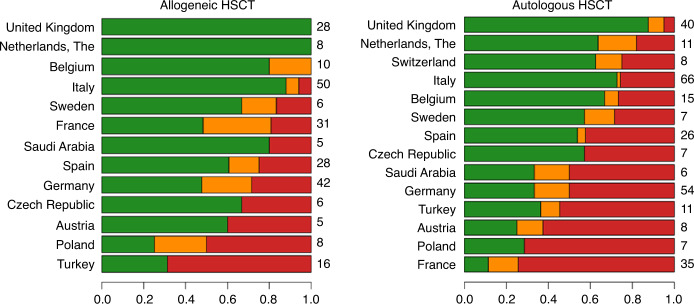
Patient Follow up per country. The bars report the percentage of reported 1-year follow-up for Centers in countries with at least 5 Centers, divided into allogeneic (left) and autologous (right) transplants in the second phase of the project (observation interval 2015–2019). On the right side of the plots, the number of Centers per country is reported. Centers with very good follow-up (>90% of transplanted patients) were classified as “Green”; Centers with a borderline completeness (80–90%) were classified as “Amber”; Centers with a Follow-up <80% were classified as “Red” and were not selected for the outcome analysis.

**Fig. 3:**
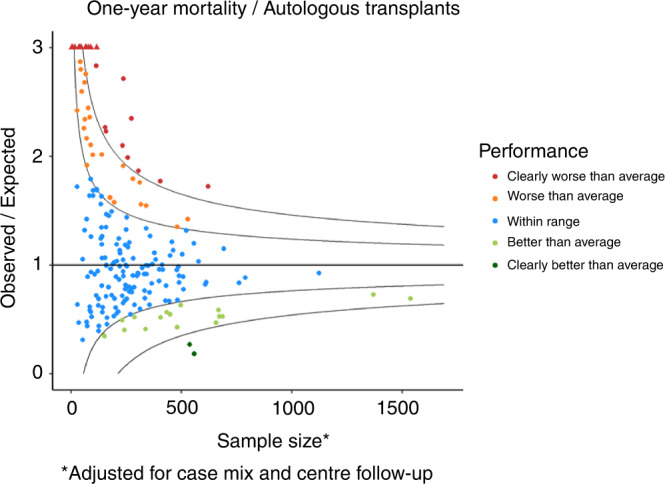
Autologous Transplants One-year mortality Funnel plot. The Funnel plot shows 1-year mortality after an autologous transplant in Centers selected for a reliable follow-up in the 2015–2019 interval (second phase) comparing observed over expected mortality, adjusted for case mix and Center follow-up.

**Fig. 4:**
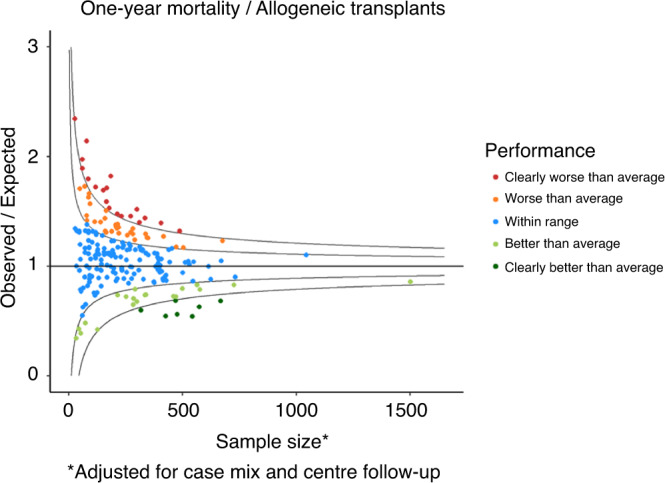
Allogeneic Transplants One-year mortality Funnel plot. The Funnel plot shows 1-year mortality after an allogeneic transplant in Centers selected for a reliable follow-up in the 2015–2019 interval (second phase) comparing observed over expected mortality, adjusted for case mix and Center follow-up.

The flow-chart of the selection process in the second phase is reported in Fig. 1. Overall, 136,320 patients out of 162,432 (83.9%) and 395 Centers out of 596 (66.3%) were selected for the analysis. The selected Centers were then analysed for 1-year follow-up benchmarking: 218 (76.8%) allogeneic Centers and 203 (60.2%) autologous Centers were eligible for the outcome analysis. Figure 2 reports the distribution of loss to 1-year FU per country. Figures 3 and 4 show the distribution in the funnel plot of Centers selected for the outcome analysis for autologous and allogeneic transplants, respectively. Each dot represents a Center, x-axis represents the effective sample size (adjusted for case mix and Center follow-up) whilst the y-axis shows the observed/expected 1-year mortality. Centers within the inner funnel are performing within range; Centers outside the outer funnel are performing worse (upper half) or better (lower half) than average, according to a multiple-testing adjusted significance level of 5%. A detailed description of the model has already been published [12].

### Report distribution

Table 1 shows the distribution of reports over the 2 phases. For the small minority of Centers who reported problems accessing a web-based drive, delivery of the report was facilitated either directly by email to the PI with a password-protected report or via the National Registries to the Center (meaning that the National Registry also accessed the report). In the first phase about half of Center PIs did not pick-up their report in spite of multiple reminders. Report pick-up improved in the second phase, probably due to improved communication and the support of the National Registries, all leading to greater awareness.

**Table 1:** Report distribution at the first and second phases.

	First phase (2013–2016)	Second phase (2015–2019)
	*N* (%)	
Total reports	268 (100)	395 (100)
No PI email	0 (0)	5 (1)
Bounced email	6 (2)	9 (2)
Successful notifications	262 (98)	381 (97)
Total Reports picked-up	127 (48)	260 (68)
Of the distributed reports	*–*	*–*
CICs accessed drive	123 (97)	200 (77)
CICs manual distribution	4 (3)	24 (9)
Distributed by National Registry	0 (0)	36 (14)
Reports not picked-up	135 (52)	121 (32)

Numbers and percentages of report distribution in the two phases of the project.

### Data completeness

**Fig. 5:**
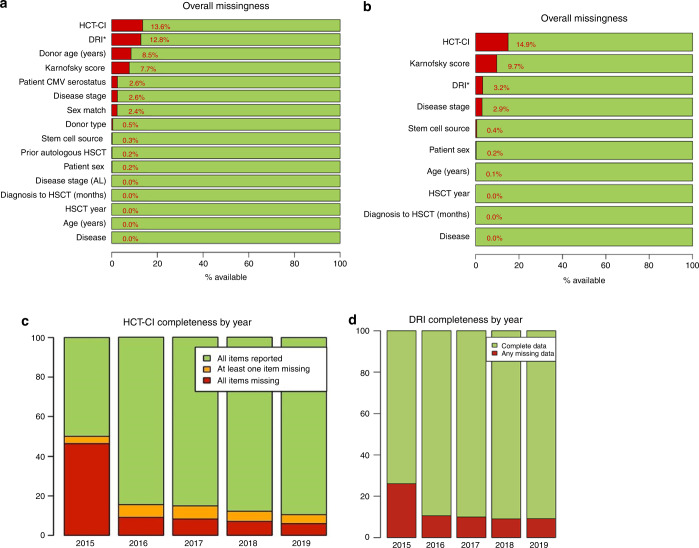
Data completeness. In the second phase of the projects (2015–2019 transplants), the lack of case-mix data is reported in allogeneic transplants (**a**) and autologous (**b**), respectively. HCT-CI was calculated based on the raw data entered in ProMISe data field; blank fields were assumed as negative. The trend to data completeness in the two composite indexes HCT-CI (**c**) and DRI (**d**) in allogeneic HSCT in the period 2015–2019 is shown in the lower panels.

Entering case-mix data in the Registry is essential for a fair and accurate benchmarking. Figure 5 reports the percentage of missing variables in the analysed cohort. For composite indices, such as HCT-CI, the bars report the frequency of missing variables, either total or partial, and the frequency of complete reports. Overall, the proportion of missing comorbidities saw a notable reduction between 2015 and 2019. The availability of HCT-CI in allogeneic transplants increased from 51.3% in 2015 to 90.9% in 2019. A similar pattern was observed in autologous transplants, in which HCT-CI availability increased from 49.9% in 2015 to 89.6% in 2019. The majority of improvement was observed between 2015 and 2016, mainly due to the introduction of new and more comprehensive MED-A forms in 2015. In allogeneic HSCT, DRI could not be calculated due to missing data in 26.1% of cases in 2015, which was reduced over time to 9.2% in 2019 (Fig. 5d). In 2015, 23.0% of undefined DRI was due to missing cytogenetics for MDS and AML, whereas in 2019, undefined DRI due to missing cytogenetics in MDS and AML was reduced to 6.7%. ‘Missingness’ due to disease stage remained stable over time, observed in 3% of patients in 2015 and 2.5% in 2019. In the benchmarking model, missing DRI was imputed as ‘Intermediate’.

**Fig. 6:**
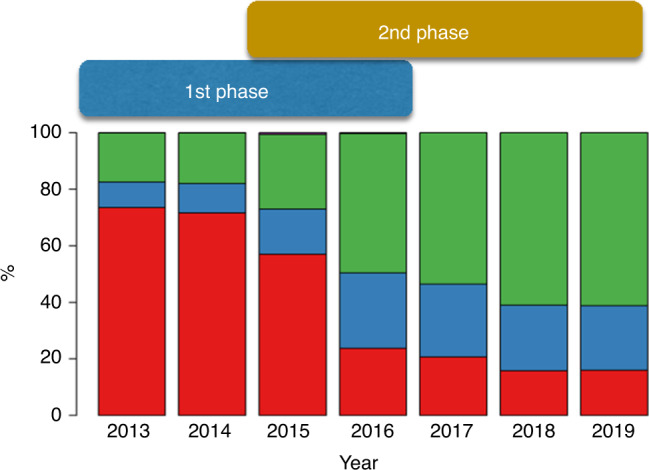
Reporting of molecular markers. The bars represent the percentage of MED-A forms reporting 1 (blue bars), more than 1 (green bars) or no molecular markers (red bars), per year of transplant. The horizontal bars above indicate the time intervals analysed in the two phases of the Benchmarking project.

Management of missing variables was described in the methods. A trend of improving data completeness was observed, such as in the case of genetic markers in AML and MDS (Fig. 6), which increasingly are being entered in the database, therefore allowing a better assessment of the prognosis [22]. A comparison of baseline data completeness in Centers included in both the analysis is also reported in Supplementary document #3, showing that the vast majority of Centers improved by >10% points.

### Center feedback

A total of 395 Centers were eligible to participate in the June/July 2021 Benchmarking exercise.

Approximately 95% of the Centers were successfully notified regarding the availability of their benchmarking report. 14 Centers could not be notified due to a lack of or problems with PI email addresses.

From the notified Centers, 68% (260 Centers) were able to pick up the report: 200 directly through Google

Drive access, 36 through their national registries and 24 were sent directly via the Helpdesk e-mail to the Centers that were not able to access the drive themselves.

Twenty-nine percent of the Centers (76) that accessed the report provided feedback. The majority (95%) of Centers that evaluated the report found the content comprehensible. Ninety-eight percent evaluated the quality as good and considered the current report an improvement on the previous release/version. Kaplan–Meier curves were considered as one of the most relevant results of the report (77% of the Centers found this most informative). Fifty-eight percent of the Centers found the funnels plots to be a relevant result for their Center. Most Centers (93%) found that the score of their program was appropriate. Ninety-eight percent of the Centers found the EBMT Benchmarking project to be relevant and of importance. Ninety-five percent of the Centers reported a positive impact on their data quality as a result of the benchmarking project.

## Discussion

The JACIE accreditation program was established by EBMT more than 20 years ago with the aim of quality improvement and ultimately better survival outcomes in HSCT patients through a system of external inspection and assessment of compliance against minimum quality standards. The positive impact of the JACIE accreditation program has been supported by a retrospective registry analysis of outcomes in allogeneic transplantation in Europe [23] with similar results also reported in North America [24]. The current and recent versions of the FACT-JACIE Standards require that Clinical Programs should achieve 1-year survival and non-relapse mortality at 100 days outcomes within or above the expected range when compared with national and international outcome data and the Center should also benchmark for non-relapse mortality at 100 days. Thus, the EBMT-JACIE benchmarking project has enabled Centers reporting to the EBMT registry to assess their performance in a validated system of comparison with the EBMT Registry, thereby providing an evidence-based evaluation for accreditation, whilst meeting the increasing clinical quality reporting demands of health authorities, regulators and payers.

There has been initial good progress in terms of both number of participating Centers and data completeness, but there are a number of issues in refinement of the EBMT benchmarking system and how it can be best delivered across all participating EBMT members (who through criteria for ‘full’ membership are required to commit to routine data submission for all transplanted patients to the EBMT registry) and applied across the range of relevant countries and their healthcare systems.

The advantages and disadvantages of EBMT-wide versus national benchmarking need to be fully considered. There is a broad range of ‘lost-to-follow-up’ between countries for both autologous and allogeneic HSCT (Fig. 2). If only Centers with high data completeness are compared with countries where a minority of Centers are included, Centers in some countries are disproportionately compared with others. It is not inconceivable that the ability to report high quality data may be related to aspects of the Center, such as size or quality of care which may impact on outcomes. Thus, an individual Center (irrespective of country) is potentially benchmarked against ‘high performers’ rather than ‘all Centers’ across the registry and potentially with only a small proportion of Centers in their own country. Some work needs to be done to address this. The simplest way is by exploring how our model works on a country-by-country basis, as well as across the whole of the EBMT. These international differences in data completeness are likely to become more pronounced with the planned increase in threshold to include only Centers completing more than 90% of data returns (i.e., green cohort). It is hoped that ongoing open publication of individual national data performance may provide impetus on a national level to enhance data completeness across individual Centers. Such open, public discussion of the importance of completeness of data at Center, national and international levels will encourage appreciation of its impact on the benchmarking process (including the need to ‘impute’ data and the potentially negative impacts on accuracy of reports if data are incomplete), hopefully supporting the concept of data managing as a primary requirement in process control. This will hopefully drive improvement in reporting across all the key variables in the benchmarking model. Clearly there are some variables that are more significant than others (e.g., 1 year survival versus other individual variables used more for risk-adaptation) and there needs to be further fine-tuning of the model in that respect. Nevertheless, the overall message should be for Centers to aim for 100% data completeness if the goal is for their outcomes assessments to be accurate and robust to not only reflect previous performance, but also for future quality improvement in patient care. It would also be a mutually useful exercise to validate the EBMT model against the various well-established national benchmarking systems which informed its development, and these are planned in the next phase of the project.

Moving forward for analysis of 2020–21 data (and potentially beyond), one very significant aspect to consider is how the variable impact of the COVID-19 pandemic is ‘fairly’ accommodated in the model. Clearly, benchmarking exercise can proceed as a ‘work in progress’, but in the exceptional times of 2020–21, during which waves of the pandemic impacted variably across geographical regions with differing socio-economic factors, there were differences in the broader public health measures and the ultimate uptake of vaccination. The full appraisal of the pandemic on HSCT patients is yet to be evaluated, but from an early stage there was recognition that outcomes following HSCT (and CAR-T cell therapy) were poorer in patients in whom SARS-2-CoV infection was detected. In addition, there was a reduction in HSCT rates, with prioritization and likely delays in treatment that may have impacted upon outcomes. Moving forward, how we accommodate COVID-19 in the model is yet to be determined, or whether a broader compensation (or even ‘amnesty’) could be based on understanding of the variable challenges between Centers and regions.

Haematology, oncology and HSCT are rapidly advancing fields which has led to ongoing development of prognostic scores which influence clinical decision making, especially in relation to high-risk treatments such as HSCT. The implementation of more comprehensive risk indices in the case-mix, aimed at improving the stratification of patients in the benchmark model is expected in the next phases of the project. This issue is strictly dependent on the availability of data in ProMISe, with special reference to the cytogenetic and molecular characterization of Acute Leukaemias (AL) and Myelodysplastic Syndromes (MDS). In our project, the Disease Risk Index (DRI) [13] was adopted, based on disease type and status at transplant, regardless of age, conditioning intensity, graft source, or donor type. DRI was validated for risk assessment in the setting of allogeneic transplantation; considering the lack of well-established prognostic indexes validated for all the diagnosis in patients undergoing an autologous transplant, it was decided to incorporate the DRI for autologous transplants too. A refinement of the DRI index, including some cytogenetic markers, was introduced in the project [12, 14] in Acute Myeloid Leukaemia (AML) and MDS; when cytogenetic data were missing, patients were classified as “intermediate” in computing the DRI score. The increased reporting of both molecular (see Fig. 4) and cytogenetic markers in the registry will allow a better stratification of patients in the years to come, possibly allowing the implementation of more powerful risk indexes in both autologous [25] and allogeneic [26] transplantation to be implemented.

The concept of “best treatment” versus “best transplant” [27] has been proposed as a goal for JACIE in the future. Increasingly HSCT is combined with post-transplant treatments, which reduce the incidence of relapse post-autologous HSCT (such as lenalidomide maintenance in myeloma) and allogeneic HSCT (with a range of evolving treatments in AL). Whilst these treatments are clearly additional to the transplant, and may often be administered by clinicians and teams outside of the BMT program, they may affect the benchmarking of outcomes in the first year and beyond. Therefore, in the foreseeable future, the model will also consider both pre-emptive and prophylactic treatments administered after the transplant in order to provide a fair clinical assessment of the process.

Moreover, there is the ongoing development and widespread use of CAR-T and other cellular therapies across a range of conditions. Such haematopoietic cellular therapies are delivered alongside HSCT by transplant and haematology programs and are subject to FACT-JACIE standards and JACIE accreditation processes. At present, the available outcomes data are insufficient to deliver a reliable benchmarking system, but with time benchmarking of CAR-T and other cellular therapies will be an essential feature of quality improvement integrated into Registry systems. Of course, the experience and learning from HSCT will be key, including the need for accurate data completeness.

Some other future developments will be the inclusion of 100-days survival, as requested by the current 8^th^ edition of the FACT-JACIE Standards, and a separate benchmarking process for adult and paediatric patient populations. EBMT is committed to equality, diversity and inclusion (EDI) and future consideration of benchmarking outcomes in relation to ethnicity, sex/gender and other characteristics may help to identify and correct health inequalities [25].

Overall, the EBMT community has been widely engaged in these early phases of the benchmarking project. The experience thus far has supported the feasibility, acceptability and reliability of the system as well as identifying some limitations. As the benchmarking model increasingly demonstrates its robustness, acceptability and relevance in modern HSCT practice (and ultimately CAR-T), there is an expectation that interest from patients and external stakeholders will grow. The benchmarking project is now a major opportunity for EBMT and JACIE with direct impact on clinical care, Center functioning and quality of survival outcomes.

## Supplementary information

Supplementary Material #1

Supplementary Material #2

Supplementary Material #3
