# Supplementary material for: Benchmarking of survival outcomes following Haematopoietic Stem Cell Transplantation (HSCT): an update of the ongoing project of the European Society for Blood and Marrow Transplantation (EBMT) and Joint Accreditation Committee of ISCT and EBMT (JACIE)
Source: Bone Marrow Transplant. 2023 Mar 9;58(6):659–66. doi: 10.1038/s41409-023-01924-6 (PMC9995719; doi:10.1038/s41409-023-01924-6)
Supplement: Supplementary file 1 — Supplementary Material #1 [file 41409_2023_1924_MOESM1_ESM.docx]

Supplemental Material #1

Trend of data completeness at baseline and 1 year follow-up between the first and second phase, divided for allogeneic and autologous HSCT.

1. **Allogeneic transplants:**

- Adjusted follow-up:

|  |  |  | **Change 2020-2022** | | |
| --- | --- | --- | --- | --- | --- |
|  | **Group** | **Not participating in both periods** | **worse** | **same** | **improving** |
|  |  |  | **N (%)** | **N (%)** | **N (%)** |
| **Total** |  |  | 49 (19.9%) | 124 (50.4%) | 73 (29.7%) |
| **Performance.2020** | Clearly worse than average | 47 (14%) | 0 (0.0%) | 25 (20.2%) | 28 (38.4%) |
|  | Worse than average |  | 3 (6.1%) | 0 (0.0%) | 9 (12.3%) |
|  | Within range |  | 5 (10.2%) | 6 (4.8%) | 23 (31.5%) |
|  | Better than average |  | 9 (18.4%) | 9 (7.3%) | 13 (17.8%) |
|  | Clearly better than average |  | 32 (65.3%) | 84 (67.7%) | 0 (0.0%) |
| **Performance.2022** | Clearly worse than average | 44 (13.1%) | 12 (24.5%) | 25 (20.2%) | 0 (0.0%) |
|  | Worse than average |  | 6 (12.2%) | 0 (0.0%) | 7 (9.6%) |
|  | Within range |  | 23 (46.9%) | 6 (4.8%) | 10 (13.7%) |
|  | Better than average |  | 8 (16.3%) | 9 (7.3%) | 14 (19.2%) |
|  | Clearly better than average |  | 0 (0.0%) | 84 (67.7%) | 42 (57.5%) |

- Adjusted follow-up in centres clearly worse than average in the first phase (2020 report):

|  |  | **N (%)** |
| --- | --- | --- |
| **Total** |  | 53 (100%) |
| **Performance in 2022** | Clearly worse than average | 25 (47.2%) |
|  | Worse than average | 7 (13.2%) |
|  | Within range | 7 (13.2%) |
|  | Better than average | 2 (3.8%) |
|  | Clearly better than average | 12 (22.6%) |

- Unadjusted follow-up change:

|  |  |  | **Change 2020-2022** | | |
| --- | --- | --- | --- | --- | --- |
|  | **Group** | **Not participating in both periods** | **worse** | **same** | **improving** |
|  |  |  | **N (%)** | **N (%)** | **N (%)** |
| **Total** |  |  | 31 (12.6%) | 156 (63.4%) | 59 (24.0%) |
| **2020** | GREEN | 47 (14%) | 30 (96.8%) | 122 (78.2%) | 0 (0.0%) |
|  | AMBER |  | 1 (3.2%) | 6 (3.8%) | 20 (33.9%) |
|  | RED |  | 0 (0.0%) | 28 (17.9%) | 39 (66.1%) |
| **2022** | GREEN | 44 (13.1%) | 0 (0.0%) | 122 (78.2%) | 47 (79.7%) |
|  | AMBER |  | 21 (67.7%) | 6 (3.8%) | 12 (20.3%) |
|  | RED |  | 10 (32.3%) | 28 (17.9%) | 0 (0.0%) |

- The greatest improvement has been observed in baseline data completeness (casemix and procedural variables, identical in 2020 and 2022 reports):

|  |  | **Not participating in both periods** | **N (%)** |
| --- | --- | --- | --- |
| **Total** |  |  | 335 (100%) |
| Data completeness (rounded percentage points, in deciles) | worse | 89 (26.6%) | 3 (1.2%) |
|  | same |  | 15 (6.1%) |
|  | improving |  | 228 (92.7%) |
|  |  |  |  |

1. **Autologous transplants:**

- Adjusted follow-up (funnel plots):

|  |  |  | **Change 2020-2022** | | |
| --- | --- | --- | --- | --- | --- |
|  | **Group** | **Not participating in both periods** | **worse** | **same** | **improving** |
|  |  |  | **N (%)** | **N (%)** | **N (%)** |
| **Total** |  |  | 61 (21.0%) | 168 (57.9%) | 61 (21.0%) |
| **Performance.2020** | Clearly worse than average | 59 (14.5%) | 0 (0.0%) | 51 (30.4%) | 29 (47.5%) |
|  | Worse than average |  | 10 (16.4%) | 1 (0.6%) | 5 (8.2%) |
|  | Within range |  | 8 (13.1%) | 4 (2.4%) | 18 (29.5%) |
|  | Better than average |  | 9 (14.8%) | 6 (3.6%) | 9 (14.8%) |
|  | Clearly better than average |  | 34 (55.7%) | 106 (63.1%) | 0 (0.0%) |
| **Performance.2022** | Clearly worse than average | 62 (15.2%) | 29 (47.5%) | 51 (30.4%) | 0 (0.0%) |
|  | Worse than average |  | 6 (9.8%) | 1 (0.6%) | 0 (0.0%) |
|  | Within range |  | 8 (13.1%) | 4 (2.4%) | 6 (9.8%) |
|  | Better than average |  | 18 (29.5%) | 6 (3.6%) | 9 (14.8%) |
|  | Clearly better than average |  | 0 (0.0%) | 106 (63.1%) | 46 (75.4%) |

- Adjusted follow-up in centres clearly worse than average in the first phase (2020 report):

|  |  | **N (%)** |
| --- | --- | --- |
| Total |  | 80 (100%) |
| Performance in 2022 | Clearly worse than average | 51 (63.7%) |
|  | Within range | 5 (6.2%) |
|  | Better than average | 4 (5.0%) |
|  | Clearly better than average | 20 (25.0%) |

- Unadjusted follow-up change:

|  | **Group** | **Not participating in both periods** | **worse** | **same** | **improving** |
| --- | --- | --- | --- | --- | --- |
|  |  |  | **N (%)** | **N (%)** | **N (%)** |
| **Total** |  |  | 28 (9.7%) | 193 (66.6%) | 69 (23.8%) |
| **2020** | GREEN | 59 (14.5%) | 18 (64.3%) | 116 (60.1%) | 0 (0.0%) |
|  | AMBER |  | 10 (35.7%) | 6 (3.1%) | 19 (27.5%) |
|  | RED |  | 0 (0.0%) | 71 (36.8%) | 50 (72.5%) |
| **2022** | GREEN | 62 (15.2%) | 0 (0.0%) | 116 (60.1%) | 58 (84.1%) |
|  | AMBER |  | 9 (32.1%) | 6 (3.1%) | 11 (15.9%) |
|  | RED |  | 19 (67.9%) | 71 (36.8%) | 0 (0.0%) |

- The greatest improvement has been observed in baseline data completeness (casemix and procedural variables, identical in 2020 and 2022 reports):

|  |  | **Not participating in both periods** | **N (%)** |
| --- | --- | --- | --- |
| **Total** |  |  | 408 (100%) |
| Data completeness (rounded percentage points, in deciles) | worse | 118 (28.9%) | 15 (5.2%) |
|  | same |  | 43 (14.8%) |
|  | improving |  | 232 (80.0%) |
