# Supplementary material for: Benchmarking of survival outcomes following Haematopoietic Stem Cell Transplantation (HSCT): an update of the ongoing project of the European Society for Blood and Marrow Transplantation (EBMT) and Joint Accreditation Committee of ISCT and EBMT (JACIE)
Source: Bone Marrow Transplant. 2023 Mar 9;58(6):659–66. doi: 10.1038/s41409-023-01924-6 (PMC9995719; doi:10.1038/s41409-023-01924-6)
Supplement: Supplementary file 2 — Supplementary Material #2 [file 41409_2023_1924_MOESM2_ESM.docx]

Supplemental Material #2

List of covariates included in the statistical model for allogeneic and autologous HSCT, respectively.

**Autologous**

| **Risk factor** | **Groups** |
| --- | --- |
| DRI | Low |
|  | Intermediate |
|  | High |
|  | Very High |
| Patient age (years) |  |
| Patient Sex | Male |
|  | Female |
| karnofsk | Moribund / Doesn`t play |
|  | Very sick / Sleeping often; play limited to passive activities |
|  | Severely disabled / Bedbound; needs assistance for quiet play |
|  | Disabled / Mainly in bed; participates in quiet activities |
|  | Requires assistance / Lying around much of the day; no active playing |
|  | Requires occasional assistance / Up and around; active play minimal |
|  | Cares for self / Greater restriction of play |
|  | Normal with effort / Active, but tired more quickly |
|  | Normal activity / Minor restrictions in strenous physical activity |
|  | Normal, NED |
| HSCT year | 2016 |
|  | 2017 |
|  | 2018 |
|  | 2019 |
|  | 2020 |

**Allogeneic**

| **Risk factor** | **Groups** |
| --- | --- |
| DRI | Low |
|  | Intermediate |
|  | High |
|  | Very High |
| Patient age (years) |  |
| karnofsk | Moribund / Doesn`t play |
|  | Very sick / Sleeping often; play limited to passive activities |
|  | Severely disabled / Bedbound; needs assistance for quiet play |
|  | Disabled / Mainly in bed; participates in quiet activities |
|  | Requires assistance / Lying around much of the day; no active playing |
|  | Requires occasional assistance / Up and around; active play minimal |
|  | Cares for self / Greater restriction of play |
|  | Normal with effort / Active, but tired more quickly |
|  | Normal activity / Minor restrictions in strenous physical activity |
|  | Normal, NED |
| Prior autologous HSCT | No |
|  | Yes |
|  | Unknown |
| Patient CMV serostatus | Negative |
|  | Positive |
|  | Not evaluated |
| HCT-CI | Low |
|  | Intermediate |
|  | High |
| HSCT year | 2016 |
|  | 2017 |
|  | 2018 |
|  | 2019 |
|  | 2020 |
| Donor age (years) |  |
| Donor type | Matched sibling |
|  | Matched other relative |
|  | Mismatched relative |
|  | Unrelated |
| Diagnosis to HSCT (months) |  |
| Diagnosis to HSCT (dummy) | 0 |
|  | 1 |
| Sex match | Male to Male |
|  | Female to Male |
|  | Male to Female |
|  | Female to Female |
| Disease stage (AL) | Not ALL or AML |
|  | ALL or AML, not in CR |
|  | ALL or AML, CR1 |
|  | ALL or AML, CR>1 |
|  | ALL or AML, CR (not specified) |
