# Supplementary material for: Benchmarking of survival outcomes following Haematopoietic Stem Cell Transplantation (HSCT): an update of the ongoing project of the European Society for Blood and Marrow Transplantation (EBMT) and Joint Accreditation Committee of ISCT and EBMT (JACIE)
Source: Bone Marrow Transplant. 2023 Mar 9;58(6):659–66. doi: 10.1038/s41409-023-01924-6 (PMC9995719; doi:10.1038/s41409-023-01924-6)
Supplement: Supplementary file 3 — Supplementary Material #3 [file 41409_2023_1924_MOESM3_ESM.docx]

Supplemental Material #3

The histograms report the baseline data completeness in Centers included in both the analysis.


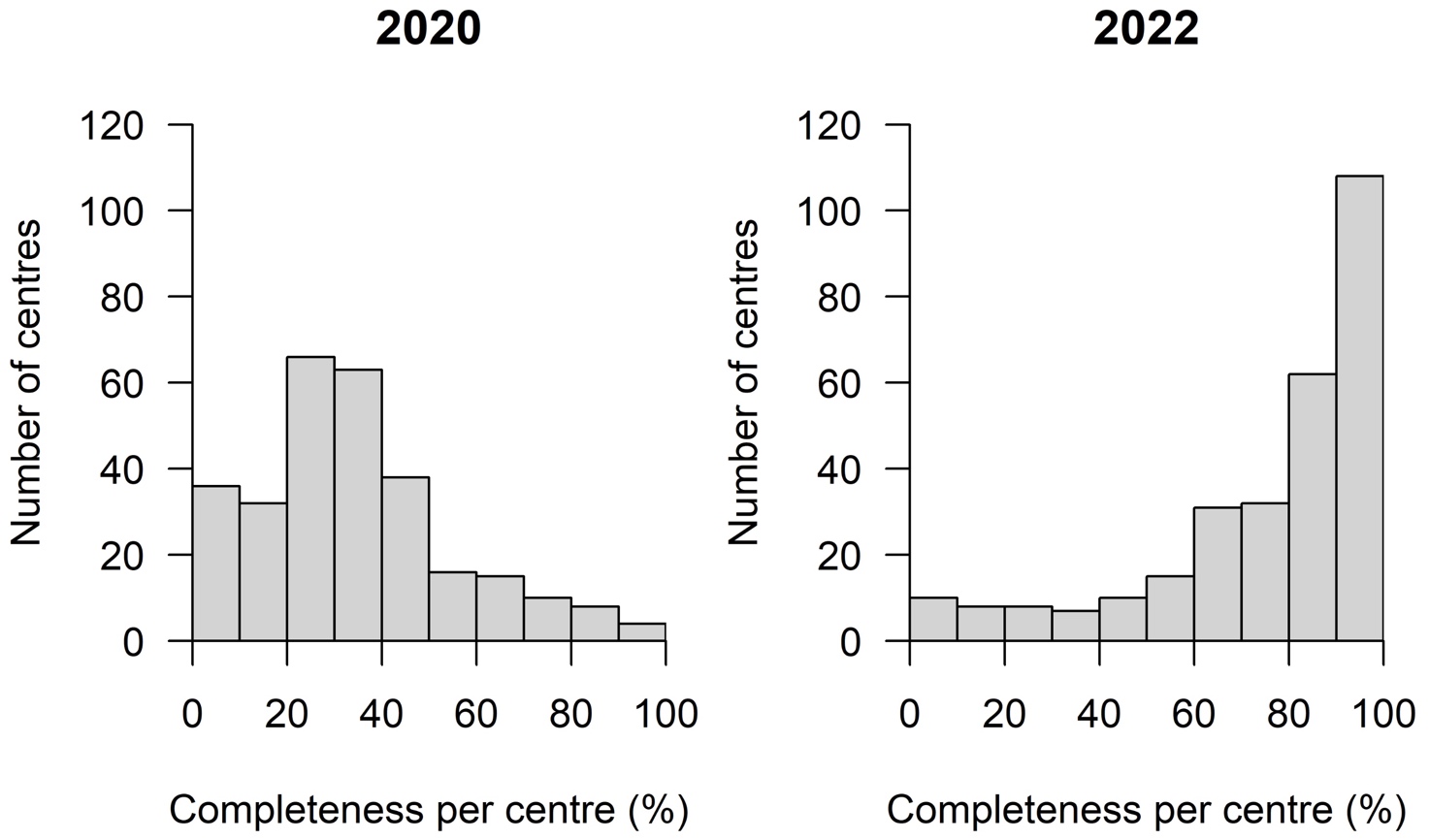


**Allogeneic Transplants**


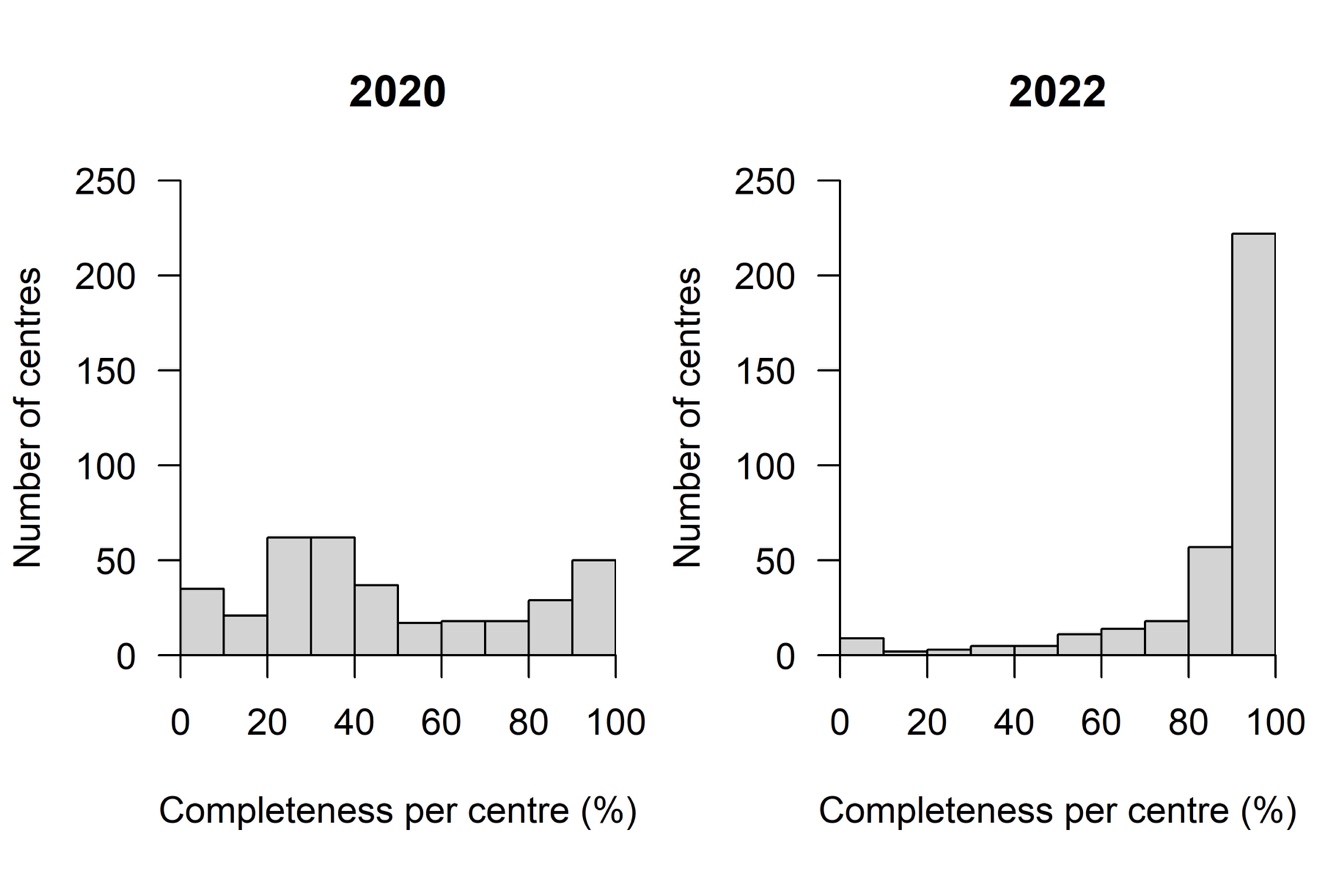


**Autologous Transplants**
